# Hybrid Sol–Gel Superhydrophobic Coatings Based on Alkyl Silane-Modified Nanosilica

**DOI:** 10.3390/polym13040539

**Published:** 2021-02-12

**Authors:** Dafna Heiman-Burstein, Anna Dotan, Hanna Dodiuk, Samuel Kenig

**Affiliations:** Department of Polymer Materials Engineering, The Pernick Faculty of Engineering Shenkar College, 12 Anna Frank Street, Ramat Gan 52526, Israel; daffyheiman@gmail.com (D.H.-B.); ADOTAN@Shenkar.ac.il (A.D.); hannad@shenkar.ac.il (H.D.)

**Keywords:** alkyl-modified hydrophilic silica, superhydrophobic coatings, sol–gel

## Abstract

Hybrid sol–gel superhydrophobic coatings based on alkyl silane-modified nanosilica were synthesized and studied. The hybrid coatings were synthesized using the classic Stöber process for producing hydrophilic silica nanoparticles (NPs) modified by the in-situ addition of long-chain alkyl silanes co-precursors in addition to the common tetraethyl orthosilicate (TEOS). It was demonstrated that the long-chain alkyl substituent silane induced a steric hindrance effect, slowing the alkylsilane self-condensation and allowing for the condensation of the TEOS to produce the silica NPs. Hence, following the formation of the silica NPs the alkylsilane reacted with the silica’s hydroxyls to yield hybrid alkyl-modified silica NPs having superhydrophobic (SH) attributes. The resulting SH coatings were characterized by contact angle goniometry, demonstrating a more than 150° water contact angle, a water sliding angle of less than 5°, and a transmittance of more than 90%. Confocal microscopy was used to analyze the micro random surface morphology of the SH surface and to indicate the parameters related to superhydrophobicity. It was found that a SH coating could be obtained when the alkyl length exceeded ten carbons, exhibiting a raspberry-like hierarchical morphology.

## 1. Introduction

The sol–gel process was initially developed to prepare ceramic materials based on silicate precursors’ solution, gelation of the solution, and finally, removal of the solvent [1,2]. The process’s simplicity led to a variety of developments using organic, inorganic, and hybrid precursors [1,2,3]. The type of precursor subsequently affects the properties of the end products. The sol–gel process’s main advantages are associated with its mild process temperatures, the variety of precursors, and the derived properties. From the chemistry point of view, the process is composed of the hydrolysis and condensation of the appropriate silicate and silane precursors into a continuous stable network.

**Figure 1:**
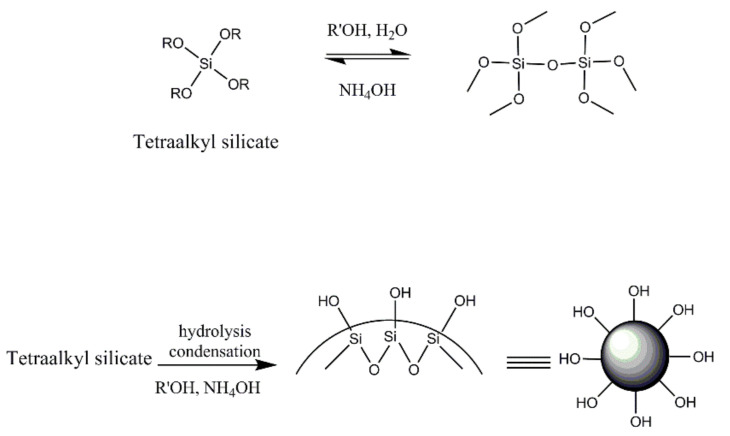
Schematic illustration of the Stöber process.

Superhydrophobic (SH) coatings based on sol–gel technology have been the subject of applied and fundamental studies [1,2,3,4], exhibiting a water contact angle (WCA) above 150° and a water sliding angle (WSA) lower than 5° [2,5]. Superhydrophobicity, as observed in nature, is characterized by a hierarchical morphology [6,7,8,9,10,11,12] comprising macro hills or pillars covered with nanometric roughness, the nature of which are three-dimensional epicuticular waxes [8,11,13,14,15] composed of long chains hydrophobic hydrocarbons. The need for roughness has been stipulated by Cassie and Baxter [2,7,16,17], supported by the fact that the WCA of the lowest surface energy of a smooth surface is 120° [13,18]. In a hierarchical structure, the contact area with the liquid is the smallest, and air is trapped between the surface asperities [13,19,20,21]. In addition to the synthesis of SH coatings, the sol–gel process can be used to prepare silica nanoparticles (NPs) by a process named after Stöber [22,23,24,25,26,27]. The Stöber process is based on a tetraalkyl silicate (TAOS) reaction with alcohol in the presence of alkaline solution and water. The end product is spherical hydrophilic silica NPs. The Stöber reaction starts with the TAOS’s hydrolysis to silanols, which condense into siloxane clusters that act as nucleates for silica NPs. By manipulating the reaction conditions, the size of the resulting NPs can be controlled [23]. The resulting silica NPs are hydrophilic by nature due to the hydroxyl groups formed on their surface, as illustrated in Figure 1.

The Stöber process can also be directly carried out on surfaces and substrates [28,29]. NPs produced in-situ are well-dispersed with fewer agglomerates. Furthermore, the formed NPs can be covalently bonded to a surface, provided that compatibility is taken care of. Though the NPs obtained by the Stöber process are hydrophilic, they can be modified through the surface hydroxyl groups. Such modifications are referred to as post modifications [30]. They are possible by introducing an appropriate reactant after the TAOS is condensed into the spherical NPs, before or after collecting the hydrophilic NPs [31,32,33,34,35,36]. The one-pot modification of the Stöber process, including the co-hydrolysis and co-condensation of tetraethyl orthosilicate (TEOS) and tridecafluorooctyl triethoxysilane (FAS), was previously reported [37]. Following the sol–gel formation, it was applied on different substrates by dipping or spraying, thus resulting in SH [38]. The sol–gel synthesized from the co-condensation and co-hydrolysis of TEOS and different silanes was applied by covering cotton and polyester fabrics. SH was achieved when using a long alkyl substitution silane and FAS. Furthermore, a third organic silane containing a non-hydrolysable epoxide group was used to increase durability. It was found that the ratio between the epoxy silane and alkyl silane is essential for SH to be achieved. The ratio between the TEOS and the silanes was not investigated. It has also been widely shown that such a modification is possible with amino-substituted silanes [39,40,41,42].

The combination of an in-situ Stöber process and one-step hydrophobic modification of the process has not been explored and could result in a straightforward, easy method to obtain SH surfaces.

Consequently, the present work aimed to explore the likelihood of modifying the hydrophilic silica NPs resulting from the conventional Stöber process to simultaneously obtain, in one step, an SH coating on a substrate via SH silica NPs and the creation of an in-situ SH coating.

The advantages of such work are clear: it is a one step process, and it creates an in-situ coating. The in-situ process would allow for better bonding to a coated surface, which might increase durability. A one step process gives better control of process parameters, and is favored by the industry.

The starting point for the study of a one-step reaction was the fact that the reactions rates of the hydrolysis and condensation of the sol–gel process are significantly influenced by the substituent groups on the silane, the solvent type, precursor concentration, and catalyst type (acidic or base) [43]. For example, it has been reported that reaction rates decrease as the alkyl substituent length increases, though there are exceptions in the case of polar substituents [43,44,45]. 

**Figure 2:**
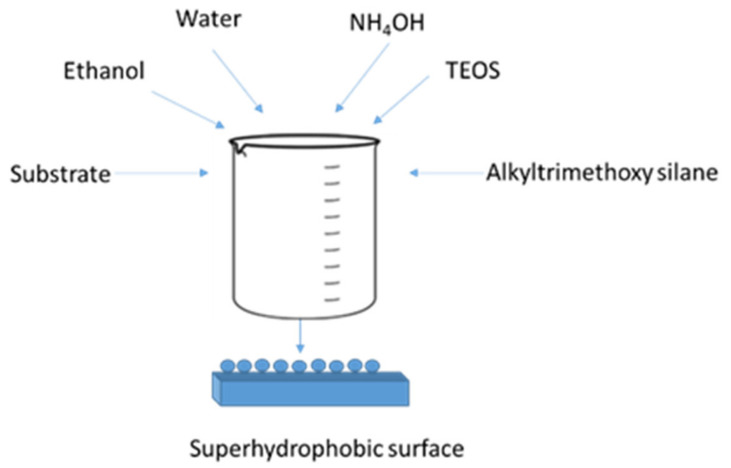
In-Situ superhydrophobic coating preparation. TEOS: tetraethyl orthosilicate.

Thus, the strategy that was followed comprised an alkyl precursor’s addition to the conventional TAOS formulations, as shown in Figure 2, to investigate the process conditions and compositions of the precursors to control reaction kinetics, though the overall influence of the alkyl substituent on the reaction rate quantification is not known. It is also not known from which alkyl length the TEOS condensation rate would exceed that of the alkyl trimethoxy silane. Accordingly, it was found that the simultaneous addition of TAOS and a low surface energy co-precursor with a long enough organic chain and appropriate concentrations led to the formation of spherical silica NPs that were superhydrophobic, with the right chemistry and morphology. Furthermore, the process could take direct place on any compatibilized substrate.

## 2. Experimental

### 2.1. Materials and Reagents

**Figure 3:**
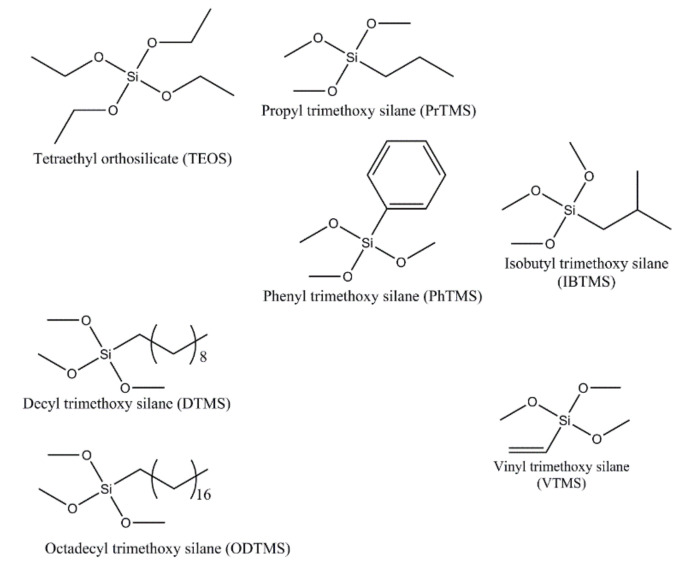
Molecular structure of the used precursors.

The following precursors were used in the study (see Figure 3): TEOS and vinyl trimethoxysilane (VTMS), which were from Sigma-Aldrich Chemie GmbH, Taufkirchen, Germany. Propyl trimethoxysilane (PrTMS), isobutyl trimethoxy silane (IBTMS), and decyl trimethoxysilane (DTMS) were from Alfa Aesar, Thermo Fisher Scientific, Heysham, Lancashire, UK. Octadecyl trimethoxysilane (ODTMS) was from J&K Scientific bvba, Lommel, Belgium. The used solvents were denatured ethanol and acetone, which were from Bio-Lab Ltd., Jerusalem, Israel. Ammonium hydroxide (28–30%) was from EMSURE^®^ Reag. Ph Eur Merck KGaA, Darmstadt, Germany. Distilled water was used for hydrolysis.

### 2.2. Pretreatment of the Substrates

Glass substrates with dimensions of 25.4 × 38.1 mm^2^ were used throughout the study. They were sonicated in acetone for 15 min and dried using paper wipes (Kimtech Science Kimwipes, Kimberly Clark, Roswell, GA, USA).

The cleaned substrates were pretreated with plasma for 2 min each side in the presence of air using low-pressure plasma (Diener electronic GMBH & CO.KG, Ebhausen, Germany).

### 2.3. Synthesis and Coating Process

The coatings were synthesized by a one-step process, as can be seen in Figure 2. The hybrid TEOS:alkyl trimethoxysilane (ATMS) molar ratio varied between 1:9 and 9:1, and the molar concentrations of the two precursors were 0.06, 0.09, 0.12, 0.18, and 0.24 M. The coatings were prepared by first adding ethanol:water at a ratio of 95:5 to the reaction vessel containing the substrate. Then, ammonium hydroxide was added to reach a pH of 10.5. Finally, TEOS and alkyl trimethoxysilane were added in the predetermined ratio and concentration. The vessel was placed on an orbital shaker and agitated overnight. The coated slides were removed, washed with ethanol, sonicated in acetone for 20 min, and then air-dried at room temperature.

### 2.4. Characterization

The WCA was measured using the sessile drop method by employing a contact angle CA device and the SCA 20 software (Data Physics, Regensburg, Germany). A 5 µL droplet of Millipore water was dropped on a horizontally coated substrate using a (Hamilton) 500 µL syringe controlled by the system. The software calculated the drop, as observed using the OCA device and the WCA values. When the drop did not adhere to the substrate and remained on the needle, the recorded WCA was >150°. The reported results are an average of three measurements taken at different locations of the substrate. The WSA was also measured using the sessile drop method with a contact angle OCA device and the SCA 20 software (Data Physics). A 30 µL droplet of Millipore water was dropped on a horizontally coated slide using a 500 µL syringe (Hamilton) controlled by the system. The device was tilted using a tilting base unit (TBU) device. The reported results are the angles in which the drop rolled off across the surface.

Haze and transmittance were measured using a haze meter (Haze-gard plus, BYK Gardner, Germany).

The surface morphology was investigated using SEM (JSM-IT200 JEOL, Japan) and a confocal microscope (µsurf expert, D-46049 Nanofocus, Germany).

## 3. Results and Discussion

### 3.1. Wettability of the Treated Surfaces

As discussed, the in-situ coatings were synthesized using TEOS and ATMS (R(SiOCH_3_)) as co-precursors. The used ATMS differed in the length of the alkyl chain (R) and the functional group, as described in Figure 3.

In the Stöber process (see Figure 1), tetraalkyl silicate is hydrolyzed by water in the presence of alcohol and ammonium hydroxide [23] and then undergoes condensation into spherical hydrophilic silica particles. By selecting the type of tetraalkyl silicate and alcohol, as well as their concentrations, particle size and reaction rates can be controlled.

In the process, ATMS is also hydrolyzed, followed by condensation. However, the result is a macromolecular network formation and not spherical silica NP growth, which is caused by the steric hindrance of the alkyl [23,37,38]. Distinctively, when alkyl silanes of a specified length and concentration are incorporated with TEOS, unexpected results may be obtained, as evidenced in Table 1. As shown in Table 1 for the case of a 0.09 M concentration of the two combined precursors, SH coatings (WCA > 150° and WSA < 5°) were obtained when incorporating ODTMS or DTMS co-precursors into TEOS. For the TEOS:ODTMS system, a wide range (7:3–4:6) of SH coatings was obtained, while in the case of the TEOS:DTMS system, an SH coating was obtained only when the ratio was 1:1. Furthermore, at ratios 6:4 and 4:6, a WCA > 150° was obtained; however, the WSA > 5°.

As can be concluded from Table 1, SH coatings were obtained in a wide concentration ratio between TEOS and DTMS or ODTMS. The other alkyl silanes did not display SH behavior. Distinctively, the SH coatings showed a high optical transmission (above 80%), but they also showed a high haze. The lowest haze for the studied system was obtained for ODTMS at a concentration ratio of 6:4 for TEOS:ODTMS. Since surface tension is commonly correlated with Hansen solubility parameters (HSPs) [46]. HSPs were calculated using the group contribution method. Surface tensions were determined (Hansen’s HSPiP software), as detailed in Table 2.

As can be deduced, the type and length of the alkyl substituents of the silane determined the resulting coating’s wettability characteristics. Faddev and McCarthy reported that alkyl chain length does not affect the wettability of a coating [48]. As shown in Table 2, the surface tensions were hardly affected by the alkyl substituent except for PhTMS, which had a higher surface tension due to the more polar nature of the phenyl compared to the linear alkyl substituents [49,50]. However, the silane substituents did affect the reactivity of the silanes themselves. This could be concluded from the attributes of the silicon atom. Accordingly, the silicon’s reactivity was affected by the negative charges that may have been induced in the transition state during the reaction. Both steric and inductive effects influenced the formation of negative transitional charges. Hence, hydrolysis was more affected by inductive effects, while steric effects had a greater influence on condensation. As a result, electron-withdrawing substituents stabilized hydrolysis, while an electron-donating reactants slowed it. For example, triorganoalkoxysilane condensation rates were decreased due to an increase in the alkyl length, as is the case for a branched alkyl substituent or a phenyl substituent [1,51]. It should be emphasized that the preferred substituents were electron-donating; thus, both electron-donating and steric effects were increased with the alkyl length increase.

The wettability of coatings is associated with surface morphology. In the case of the Stöber silica formation process, the co-precursors’ change could facilitate or restrict the formation of silica NPs by the TEOS constituent, which dominated the surface morphology.

**Figure 4:**
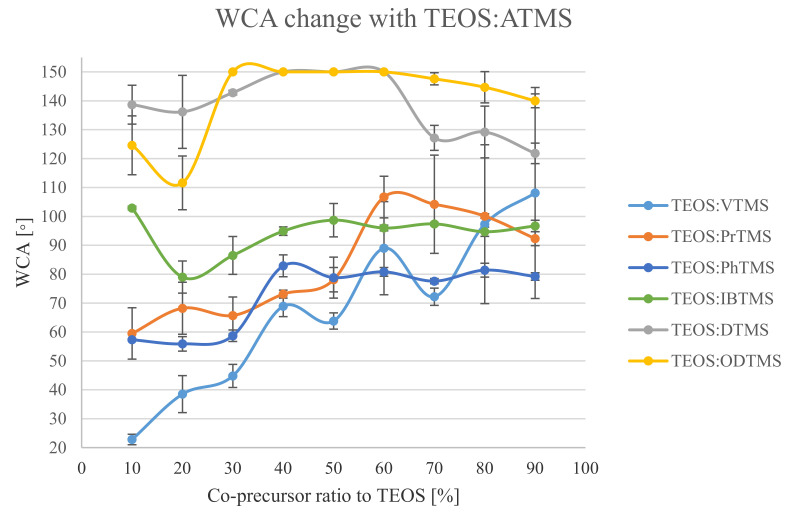
WCA change with co-precursor type and ratio.

Figure 4 demonstrates that at a low co-precursors’ ratio of TEOS and various alkyl silanes, the WCA could be divided into two groups. The first group possessed long alkyl substituent ATMS, and the other group possessed short alkyl substituent ATMS. In the long alkyl substituents (DTMS and ODTMS), silica NPs were formed at all ratios, thus resulting in higher WCAs. These results were related to the stoichiometric ratio between the hydroxyl groups on the silica NPs and the alkyl silanols that were available for reaction. When the ratio of the long alkyl substituent ATMS increased, the system reached a point where the developing morphology combined with the low surface tension alkyl co-precursor resulted in an SH coating. Moreover, when the ATMS:TEOS ratio was increased, the morphology formation was disturbed.

In short alkyl ATMS with low ratios of the co-precursor, the WCAs were mainly controlled by the TEOS. Additionally, when increasing the co-precursor concentration, an increase in the WCA was observed, to the point where the ATMS co-precursor was the dominant factor. Distinctively, the incorporation of the VTMS co-precursor resulted in a non-uniform coating, especially with the high VTMS ratios. This was probably due to the phase separation that manifested in large standard deviations of the measured WCA, ranging from 27.5 to 36.5°. The other used co-precursors were IBTMS, PrTMS, and PhTMS, and they demonstrated their surface tension effects in high ratios. The lowest obtained WCAs were for PhTMS, which had the highest surface tension. The highest WCAs were obtained with IBTMS system, which had the lowest surface tension.

### 3.2. Morphology of the Coatings

**Figure 5:**
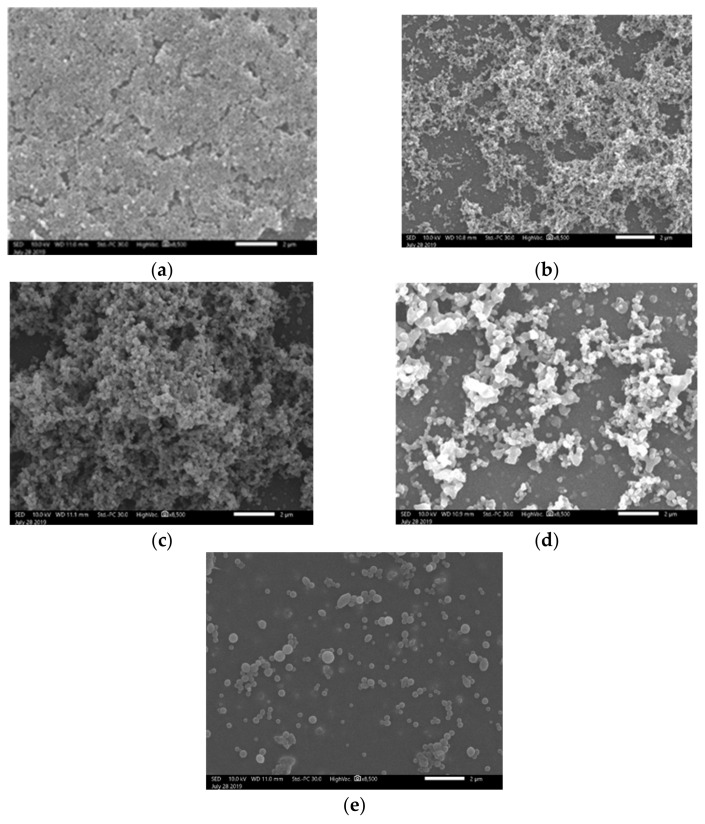
SEM images of coatings based on TEOS:DTMS ratios: (**a**) 7:3, (**b**) 6:4, (**c**) 5:5, (**d**) 4:6, and (**e**) 3:7. Bar size—2 µm

**Figure 6:**
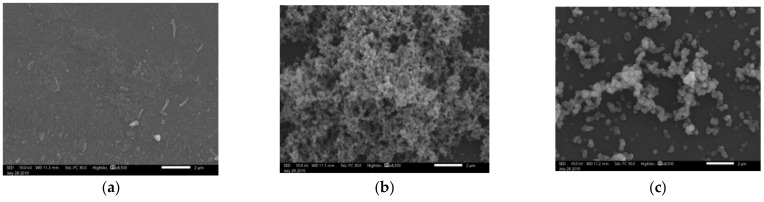
SEM images of coatings composed of: (**a**) TEOS:PrTMS, (**b**) TEOS:DTMS, and (**c**) TEOS:ODTMS at a molar ratio of 5:5. Bar size—2 µm

Table 3 summarizes the average particle size, as seen in the SEM micrographs. With an increase of the DTMS concentration concerning TEOS, the particle size (and standard deviations) also increased. In Figure 5d, platelet rather than sphere formation can be observed. This may be attributed to the excess of DTMS in the system, which condensed to form platelets. This is further substantiated in Figure 5e, where it can be seen that DTMS further increased, large particles covered the majority of the surface, and spherical particles were found to cover the coating. It can be seen in Figure 6a that spherical particles were not formed. This may be attributed to the low concentration of DTMS that may have reacted with the TEOS, thus inhibiting the formation of spherical NPs.

Figure 6 shows the coatings’ morphology in the cases of PrTMS, DTMS, and ODTMS co-precursors with increasing alkyl lengths at a molar ratio of 5:5 with respect to TEOS. As is evident from Figure 6, spherical particles were not formed in the case of PrTMS, since the TEOS constituent did not condense to form silica NPs. When the alkyl substituent length increased to 10 carbons (DTMS) or 18 carbons (ODTMS), the formation of spherical NPs with different particles sizes was achieved, as displayed in Table 4. Thus, it could be deduced that the formation of silica NPs and SH morphology are related to the length of the alkyl substituent and the resulting steric hindrance. When the alkyl substituent length is insufficient, the condensation of the alkyl trimethoxy silane and TEOS would likely occur simultaneously, which would result in coating without particles, as is evident in Figure 6a.

### 3.3. Confocal Morphology Analysis

Though confocal microscopy is limited to the micron–submicron range, it is able to shed light on the morphology of random surfaces. The numerical data obtained by the confocal microscopy could be used for the statistical analysis of the surface morphology. Table 5 summarizes the main statistical parameters of the various studied systems.

**Figure 7:**
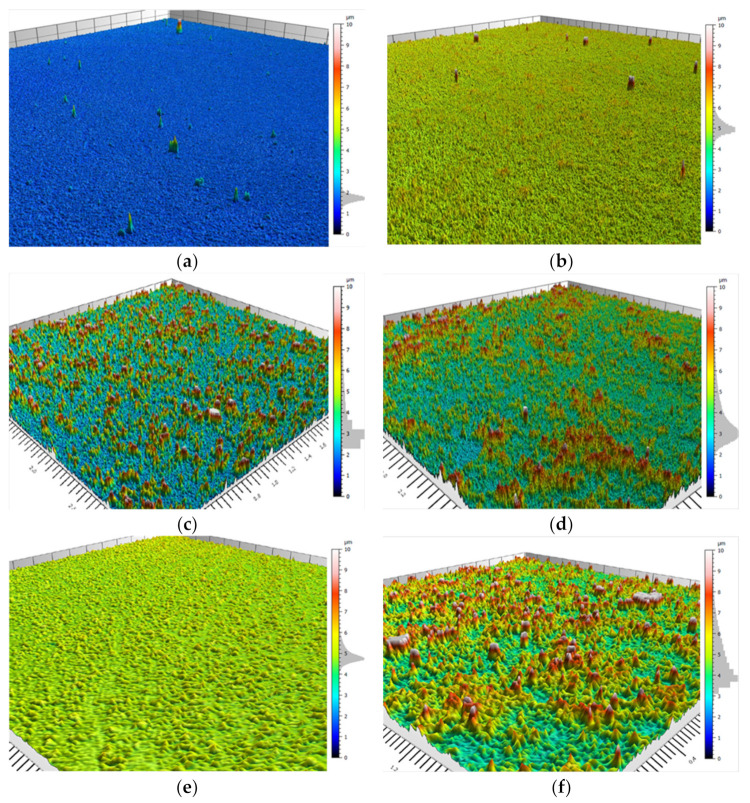

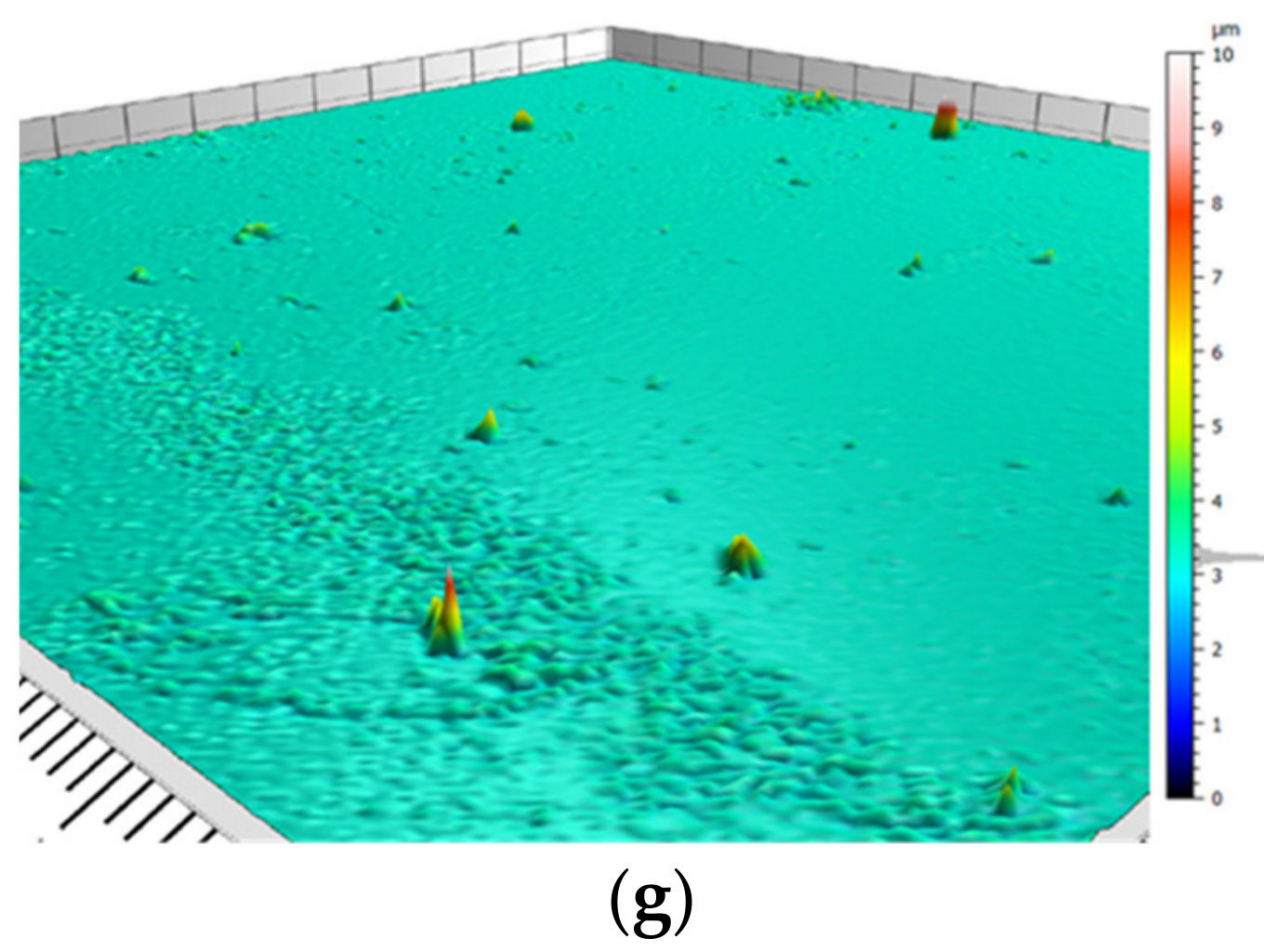
Confocal microscopy of (**a**) TEOS:DTMS at a ratio of 7:3, (**b**) TEOS:DTMS at a ratio of 6:4, (**c**) TEOS:DTMS at a ratio of 5:5, (**d**) TEOS:DTMS at a ratio of 4:6, (**e**) TEOS:DTMS at a ratio of 3:7, (**f**) TEOS:ODTMS at a ratio of 5:5, and (**g**) TEOS:PrTMS at a ratio of 5:5. Scale in µm.

Rq is commonly used to characterize surface roughness and is an indication of statistical surface height. A higher Rq value means a rougher surface, while a lower Rq value indicates a smoother surface [16,52,53]. As can be noticed in Table 5 the Rq values varied in the different systems. For TEOS:DTMS ratios of 7:3, 6:4, and 3:7 and the TEOS:PrTMS ratio of 5:5, the value was close to zero, thus indicating a smooth surface—as is also indicated by the SEM images in Figure 5a,b,e) which reveals smooth coatings that are visually demonstrated in Figure 7. Since roughness is essential for SH coatings [52,54]^,^ these coatings are not SH; it is evident when Rq values exceed 0.8 µm, SH is obtained.

As can be noticed in Table 4, the micron size asperities and SH were obtained in two systems: TEOS:DTMS at a ratio of 5:5 (2.13 ± 0.16 µm) and TEOS:ODTMS at a ratio of 5:5 (2.21 ± 0.29 µm). Other studied DTMS systems approached and exceeded the micron size, but their roughness was lower due to the higher peak density. These findings support the conclusion that the main differences in the coatings are based on different TEOS:DTMS concentrations.

Surface skewness (Rsk) is an indication of roughness asymmetry. As such, a surface exhibiting a Gaussian height distribution would have an Rsk of zero [55]; in all systems, the skewness was positive, thus indicating random peaks and asperities [56]. Surface kurtosis (Rku) is an indication of the sharpness of a height distribution. A surface exhibiting a Gaussian distribution will have an Rku of 3 [55]. As manifested in Table 5, an Rku > 3 was obtained in all systems. As pointed out by Nahum et al. [52], skewness and kurtosis are not characteristics for SH, as also shown in this work.

Confocal microscopy is generally limited to micro-sized roughness, while scanning electron microscopy allows for the sub-micron analysis of a surface. Thus, in Figure 7, micro asperities, as opposed to the nanometric asperities displayed in Figure 5 and Figure 6, can be observed. As stated before, both micro-and nanometric roughness are essential for hierarchal morphology to obtain SH coatings. As seen in Figure 7a,g, TEOS:DTMS at a ratio of 7:3 and TEOS:PrTMS at a ratio of 5:5, respectively, obtained almost smooth surfaces. When examining coatings of TEOS:DTMS ratios of 5:5 and 4:6 (as seen in Figure 7c,d, respectively), a rough surface was observed, while for ratios of 6:4 and 3:7 (as seen in Figure 7b,e, respectively), the surface was more uniform. The statistical analysis parameters (Table 5) indicated that the roughness was not sufficient to achieve SH. When comparing Figure 7c,d, it can be seen that the latter showed a high level of standard deviations (Table 3) and the confocal surface image resembled agglomerated particles, which might explain the WCA > 150° and the WSA > 10°, which is not considered to comprise SH.

### 3.4. Reaction Mechanisms

**Figure 8:**
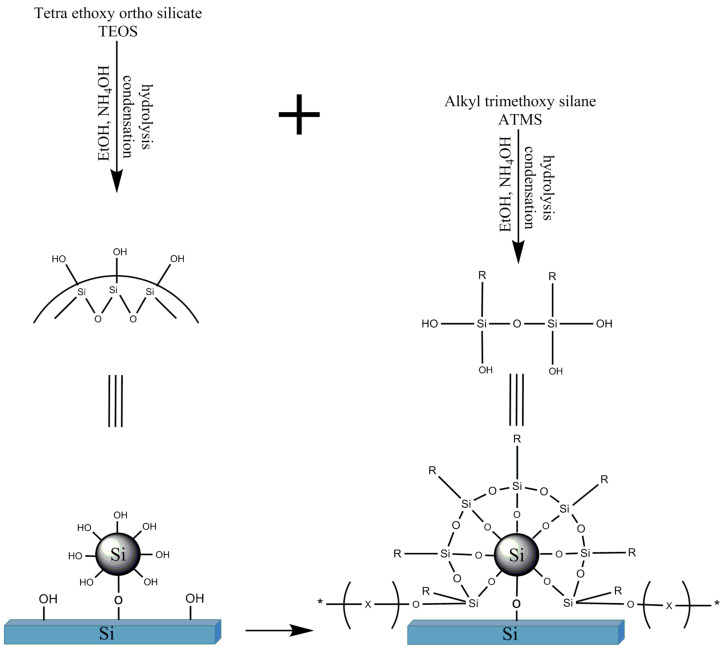
Proposed reaction mechanism.

As discussed by Brinker et al. [1], Osterholtz [23], and Pohl [25], the condensation of ATMS is slower than that of TEOS due to the silane alkyl group steric hindrance. Thus, the longer the silane alkyl substituent, the more significant the steric hindrance and the slower the condensation. As a result, the TEOS condensation reaction may proceed undisturbed to form hydrophilic silica NPs according to the well-known Stöber reaction. The process can also be reffered to as co-hydrolosis and co-condensation, as proposed by Wang et al. [37,38]. Based on the results mentioned above and the cited literature, a proposed mechanism was hypothesized, as depicted in Figure 8. The proposed mechanism is supported by the results shown in Figure 6b,c, exhibiting the steric hindrance effect of decyl and octadecyl alkyl chains that allowed for the formation of silica NPs. When the alkyl substituents were short and did not cause a steric hindrance, the self-condensation of the co-precursors (alkyl silanes) took place simultaneously with the TEOS constituent. In this case, no silica NPs were formed. It is evident from Figure 6a that when the co-precursor used was PrTMS, the propyl substituent inhibited the formation of spherical NPs. Distinctively, SH coatings were accomplished when silica NPs decorated with the alkyl silanes with a low surface tension were obtained. As suggested in Figure 8, the hydrophilic hydroxyl groups covering the external surface of the silica NPs obtained by the classic Stöber process reacted with the silanols of the hydrophobic alkyls to form SH coatings. Following these arguments, the stoichiometry of the hydroxyl groups and silanols had to be adjusted accordingly. Since the long alkyl substituent tended to fold, there may be some cases where complete stoichiometry is not achieved in the formation of SH coatings. One should note the WCA values in Table 1 for the TEOS:DTMS system when considering the latter result. In this system, the alkyl substituent was composed of 10 carbons, and superhydrophobicity was achieved in a limited range of concentrations. However, in the TEOS:ODTMS system where the alkyl substituent had 18 carbons, superhydrophobicity was achieved in a wide range of precursors molar ratios.

## 4. Conclusions

Hybrid sol–gel superhydrophobic coatings were obtained based on the classic Stöber reaction, which was modified by the co-addition of long-chain alkyl silanes, for the preparation of hydrophilic silica NPs. The reaction took place directly on the compatibilized substrate. SH coatings were obtained by combining sol–gel chemistry comprising TEOS and various ATMS, which differed in the type and length of the alkyl substituent. The SH coatings were obtained when the alkyl substituent was long enough to cause steric hindrance to slow down its self-condensation. In this way, the formation of the hydroxyl-functionalized spherical silica NPs via the TEOS moiety was realized, followed by the condensation of the alkyl silanols with the NP hydroxyl groups. As the silica NPs directly condensed on the substrate surface, covalent bonding between the TEOS/alkyl silane systems and compatibilized (oxygen-treated) substrates led to the covalent bonding of the silane-treated particles to the substrate. It was established that when the alkylsilane chain length was above 10 carbons and there was an appropriate molar concentration ratio between the TEOS and the alkyl silane, an SH coating with a raspberry-like hierarchal morphology was obtained. This straightforward approach could be extended to prepare SH coatings with other hydrophobic co-precursors like fluoroalkyl silanes and combinations of co-precursors to increase the coating’s durability.

## Figures and Tables

**Table 1:** Wettability and optical properties of coatings using various precursors concentration of 0.09 M. VTMS: vinyl trimethoxysilane. ODTMS: octadecyl trimethoxysilane; WCA: water contact angle; WSA: water sliding angle; VTMS: vinyl trimethoxysilane; PrTMS: propyl trimethoxysilane; DTMS: decyl trimethoxysilane; IBTMS: isobutyl trimethoxy silane.

TEOS:VTMS	WCA Avg. (°)	WSA (°)	Haze (%)	Transmittance (%)	TEOS:PrTMS	WCA Avg. (°)	WSA (°)	Haze (%)	Transmittance (%)
9:1	22.8 ± 1.8	>90.0	2.5	93.8	9:1	59.5 ± 8.9	90.0	0.2	93.3
8:2	38.5 ± 6.4	>90.0	1.7	96.8	8:2	68.2 ± 9.0	>90.0	1.6	93.0
7:3	44.8 ± 4.0	>90.0	1.7	95.4	7:3	65.7 ± 6.4	>90.0	0.7	93.0
6:4	68.9 ± 3.6	>90.0	2.0	95.6	6:4	73.1 ± 1.4	>90.0	1.4	93.1
5:5	63.8 ± 2.8	>90.0	2.1	92.8	5:5	78.1 ± 4.2	90.0	0.3	92.8
4:6	89.0 ± 16.1	>90.0	4.4	93.7	4:6	106.7 ± 7.2	>90.0	6.0	91.6
3:7	72.2 ± 3.0	>90.0	6.3	94.2	3:7	104.2 ± 17.0	40.0	40.0	88.8
2:8	97.3 ± 27.5	>90.0	8.5	92.9	2:8	100.1 ± 0.8	50.0	30.8	91.1
1:9	108.1 ± 36.5	>90.0	66.0	64.8	1:9	92.3 ± 2.4	27.0	6.7	92.7
**TEOS:DTMS**	**WCA Avg. (°)**	**WSA (°)**	**Haze (%)**	**Transmittance (%)**	**TEOS:ODTMS**	**WCA Avg. (°)**	**WSA (°)**	**Haze (%)**	**Transmittance (%)**
9:1	138.7 ± 6.7	73.0	36.4	93.7	9:1	124.6 ± 10.2	70.0	30.3	93.1
8:2	136.2 ± 12.6	90.0	25.4	94.2	8:2	111.6 ± 9.3	38.0	37.5	89.9
7:3	142.8 ± 0.8	67.0	22.1	95.1	7:3	150.0 ± 0.0	0.0	82.8	88.1
6:4	150.0 ± 0.0	10.0	49.5	90.1	6:4	150.0 ± 0.3	5.0	35.6	91.7
5:5	150.0 ± 0.0	3.0	82.3	81.4	5:5	150.0 ± 0.0	0.0	81.0	84.8
4:6	150.0 ± 0.0	18.0	95.2	67.2	4:6	150.0 ± 0.0	2.0	53.6	89.9
3:7	127.2 ± 4.3	50.0	66.1	88.8	3:7	147.6 ± 2.1	30.0	64.2	83.8
2:8	129.2 ± 8.9	85.0	65.3	91.9	2:8	144.7 ± 5.4	42.0	4.1	77.8
1:9	121.8 ± 3.5	90.0	76.9	92.8	1:9	140.0 ± 2.4	30.0	81.6	83.7
**TEOS:PhTMS**	**WCA Avg. (°)**	**WSA (°)**	**Haze (%)**	**Transmittance (%)**	**TEOS:IBTMS**	**WCA Avg. (°)**	**WSA (°)**	**Haze (%)**	**Transmittance (%)**
9:1	57.3 ± 0.3	>90.0	2.0	93.2	9:1	102.9 ± 0.61	30.0	4.5	96.5
8:2	55.9 ± 2.5	>90.0	2.5	93.4	8:2	79.0 ± 5.57	75.0	0.1	95.5
7:3	58.7 ± 2.0	>90.0	1.3	93.4	7:3	86.5 ± 6.56	>90.0	1.5	95.1
6:4	82.9 ± 3.8	>90.0	0.4	93.5	6:4	94.9 ± 1.47	69.0	6.7	95.0
5:5	78.8 ± 7.1	>90.0	3.4	93.8	5:5	98.7 ± 5.77	48.0	2.4	93.8
4:6	80.8 ± 1.5	>90.0	0.7	93.2	4:6	96.0 ± 1.0	32.0	1.0	93.4
3:7	77.6 ± 0.9	>90.0	1.4	93.1	3:7	97.4 ± 0.55	49.0	0.8	93.1
2:8	81.4 ± 2.4	>90.0	2.2	93.2	2:8	94.7 ± 1.59	33.0	0.2	93.3
1:9	79.2 ± 1.3	>90.0	2.1	93.4	1:9	96.7 ± 1.97	25.0	1.7	93.1

**Table 2:** Calculated surface tension from HSPiP software [47].

Precursor	Surface Tension (mN/m)
PrTMS	18.1
VTMS	18.6
PhTMS	26.0
DTMS	19.2
ODTMS	19.8
IBTMS	17.1

**Table 3:** Average particle size measured from the SEM images in Figure 5 at the following TEOS:DTMS ratios: Figure 5a: 7:3; Figure 5b: 6:4, Figure 5c: 5:5; Figure 5d: 4:6; and Figure 5e: 3:7.

Image	Average Particle Size (nm)
Figure 5a	No particles
Figure 5b	110 ± 12
Figure 5c	148 ± 18
Figure 5d	375 ± 216
Figure 5e	455 ± 267

**Table 4:** Average particle size determined from SEM images of Figure 6: Figure 6a: TEOS:PrTMS; Figure 6b: TEOS:DTMS; and Figure 6c: TEOS:ODTMS at a molar ratio of 5:5.

Image	Average Particle Size (nm)
Figure 6a	No particles
Figure 6b	148 ± 18
Figure 6c	370 ± 75

**Table 5:** Confocal microscopy morphological characteristics, where Rp (µm) is the maximum peak height of the roughness profile, Rq (µm) is the root-mean-square (RMS) deviation of the roughness profile, Ra (µm) is the arithmetic mean deviation of the roughness profile, RSm (mm) is the mean width of the roughness profile elements, peak density (1/cm) is the peak count of the roughness profile, Rsk is the skewness of the roughness profile, and Rku is the kurtosis of the roughness profile.

Parameter/ System	TEOS:DTMS 7:3	TEOS:DTMS 6:4	TEOS:DTMS 5:5	TEOS:DTMS 4:6	TEOS:DTMS 3:7	TEOS:ODTMS 5:5	TEOS:PrTMS 5:5
Rp (µm)	0.37 ± 0.07	0.90 ± 0.12	2.13 ± 0.16	1.68 ± 0.25	0.92 ± 0.09	2.21 ± 0.29	0.23 ± 0.08
Ra (µm)	0.12 ± 0.02	0.25 ± 0.03	0.63 ± 0.07	0.50 ± 0.09	0.25 ± 0.03	0.67 ± 0.08	0.06 ± 0.02
Rq (µm)	0.15 ± 0.02	0.32 ± 0.05	0.81 ± 0.08	0.63 ± 0.11	0.32 ± 0.03	0.85 ± 0.11	0.08 ± 0.03
RSm (mm)	0.01 ± 0.00	0.01 ± 0.00	0.02 ± 0.01	0.02 ± 0.00	0.01 ± 0.00	0.02 ± 0.01	0.03 ± 0.03
Peak density (1/cm)	1135 ± 156	815 ± 121	480 ± 74	647 ± 74.1	719 ± 54.8	598 ± 118	483 ± 175
Rsk	0.0112 ± 0.101	0.54 ± 0.01	0.71 ± 0.11	0.63 ± 0.11	0.63 ± 0.14	0.51 ± 0.14	0.20 ± 0.26
Rku	3.10 ± 0.28	3.72 ± 0.24	3.65 ± 0.28	3.48 ± 0.26	3.97 ± 0.38	3.30 ± 0.32	5.30 ± 0.78

## Data Availability

No data availability.
